# Exploring the Efficacy of Ketamine as an Anesthetic and Antidepressant in Postpartum Depression: A Case Study Analysis

**DOI:** 10.7759/cureus.55208

**Published:** 2024-02-29

**Authors:** Clara Benjamin, Rediet Tefera Atalay, Oluwapelumi Kolawole, Miguel Ramallo, Valerie McAllister, Oluwasegun A Akinyemi, Mahlet Siraga, Miriam B Michael

**Affiliations:** 1 Internal Medicine, Howard University College of Medicine, Washington, D.C., USA; 2 Internal Medicine, Howard University Hospital, Washington, D.C., USA; 3 Health Policy and Management, University of Maryland School of Public Health, College Park, USA; 4 Surgery, Howard University College of Medicine, Washington, D.C., USA; 5 Internal Medicine, University of Maryland, College Park, USA

**Keywords:** anti-depressants, maternal mental health, anesthesia, pregnancy, postnatal depression, postpartum depression, ketamine

## Abstract

Postpartum depression is a common mental health disorder that affects women within six months after giving birth. It is characterized by sadness, anxiety, and extreme fatigue, which can significantly impact a woman’s daily functioning and ability to care for her newborn. While traditional treatments for postpartum depression include therapy and medication, recent studies have shown promising results using ketamine. We present a case of a woman with a history of depression who delivered four children by cesarean section with debilitating postpartum depression in two births and no symptoms of depression in the births where she received ketamine during delivery.

## Introduction

Postpartum depression (PPD) is a common mental health disorder that affects women within six months after giving birth. However, the onset can be in the peripartum period as well. The American Psychiatric Association’s Diagnostic and Statistical Manual of Mental Disorders, Fifth Edition, (DSM-5) recognizes PPD also as the peripartum onset of the major depressive disorder occurring during or within four weeks following pregnancy. It is characterized by a loss of interest in activities, depressed mood, sleep disturbance, lack of energy, irritability, anxiety, and thoughts of suicide [1].

Although the exact cause of PPD is not clearly understood, it is thought to be caused by the fluctuations in reproductive hormones after childbirth [2]. The prevalence of PPD is 14% worldwide and was found to be higher among primiparous women when compared to multiparous women [3,4]. PPD is diagnosed using different tools, including the Edinburgh Postnatal Depression Scale (EPDS) and the Beck Depression Inventory [5]. Several risk factors for PPD have been identified, including prior history of depression, prenatal anxiety or depression, low social support, maternity blues, and delivery complications [6,7]. While traditional treatments for postpartum depression include therapy and medication, recent studies have shown promising results using ketamine [8].

This article was earlier presented as a poster at the Society of Ob/ Gyn Hospitalists, Chicago, IL, on September 9, 2023.

## Case presentation

A 31-year-old multiparous female was seen in the clinic following her delivery by scheduled C-section of her fourth child. She presented with psychomotor agitation and disorganized behavior on postpartum day 30. The patient had a history of mild intermittent asthma and depression. The first episode of depression was at age 22 and lasted 18 weeks, and according to the patient, seemed to improve with deliberate heavy exercise and multiple hours of sun exposure. Her family history is significant only for depression on her maternal side and a strong history of schizophrenia with numerous first and second cousins and an uncle with active disease. She experienced a similar episode at age 25 after delivering her first child, which resolved after 16 weeks without treatment. She subsequently had two more pregnancies. In her second pregnancy, the patient was given ketamine to achieve adequate analgesia. During her third pregnancy, the patient experienced heavy vaginal bleeding and hypotension after four hours of labor, necessitating an emergency cesarean section. Initial epidural anesthesia proved inadequate for pain management, leading to the supplementary administration of ketamine. Following both the second and third deliveries, in which ketamine was used, the patient demonstrated positive postpartum adaptation. She successfully bonded with her newborns, established breastfeeding, and was able to return to work within two weeks.

At admission, the patient presented with routine diagnostic assessments, including physical examination and laboratory studies, which were normal. The patient scored 24/30 on the EPDS, a 10-item self-report scale (each question scored from 0 to 3) designed to identify women experiencing depressive symptoms (cutoff score to identify a woman as depressive: 13). Antidepressive medication therapy was proposed earlier but rejected by the patient and her family, with the patient agreeing to get treatment if symptoms did not improve after four weeks. The patient started antidepressant medication four weeks later, and in the second week after starting medication, improvement was observed in which the psychomotor agitation disappeared. Slight somnolence and time and space disorientation could still be observed. Four weeks after the initiation of treatment, all symptoms had vanished, and treatment was stopped three months later.

The patient’s prenatal course was uneventful, and her routine supplement regimen included daily iron, folate, and a multivitamin. Laboratory results, urinalysis, blood pressure, and other vital signs were within normal limits throughout her prenatal visits. All four childbirths were by C-section. The first childbirth was due to fetal distress, accompanied by postpartum depression (Table 1). Epidural anesthesia was administered. The second childbirth, preceded by 23 hours of labor, was complicated by fetal distress, hypotension, and increased asthma symptoms evidenced by audible maternal wheezing. Ketamine (300 mg IV bolus followed by 0.2 mg/min infusion) was administered, resulting in visual and auditory hallucinations lasting 12 hours post surgery. On discharge, the patient had no symptoms or feelings of depression or dysthymia. The third childbirth was complicated by maternal hemorrhage and hypotension four hours into labor. Ketamine supplemented with midazolam (300 mg IV bolus followed by 0.2 mg/min infusion) was administered. The patient experienced transient visual and auditory hallucinations for a few hours post surgery but reported no symptoms or feelings of depression or dysthymia upon discharge. The fourth childbirth was a C-section with epidural anesthesia, followed by postpartum depression.

**Table 1:** Birth details of the four children

Child	Gender	Birth Weight (lbs, oz)	Birth Length (inches)	Apgar Score	Age of Mother (years)
1	Female	7 lbs, 3 oz	20	9	26
2	Female	8 lbs, 2 oz	19	10	28
3	Male	6 lbs, 10 oz	20	10	29
4	Male	7 lbs, 6 oz	21	9	31

## Discussion

Depression during the postpartum period is not uncommon, with prevalence rates as high as 20% (and perhaps even more prevalent in low-income countries) [3]. It is the leading cause of mortality during the postpartum period in England and second in the United States after homicide [9,10]. PPD is the onset of major depressive disorder occurring during or within four weeks following pregnancy. Because of the profound physiological changes that characterize pregnancy and childbirth, especially the dramatic changes in steroid hormone levels, and because of the perturbations in mood, appetite, energy, and sleep associated with childbirth and infant care, it has been argued that some PPDs are different than depressions that occur at other times.

To address questions regarding the comparability of depression symptoms experienced in the postpartum period and depression symptoms experienced outside of the postpartum period, Buttner et al. [11] and O’Hara et al. [12] evaluated the symptom structure of depression in populations of postpartum and nonpostpartum women and found that the overall structure of depression in postpartum and nonpostpartum women seems to be remarkably similar to major depression that occurs at other times in a woman's life.

**Figure 1:**
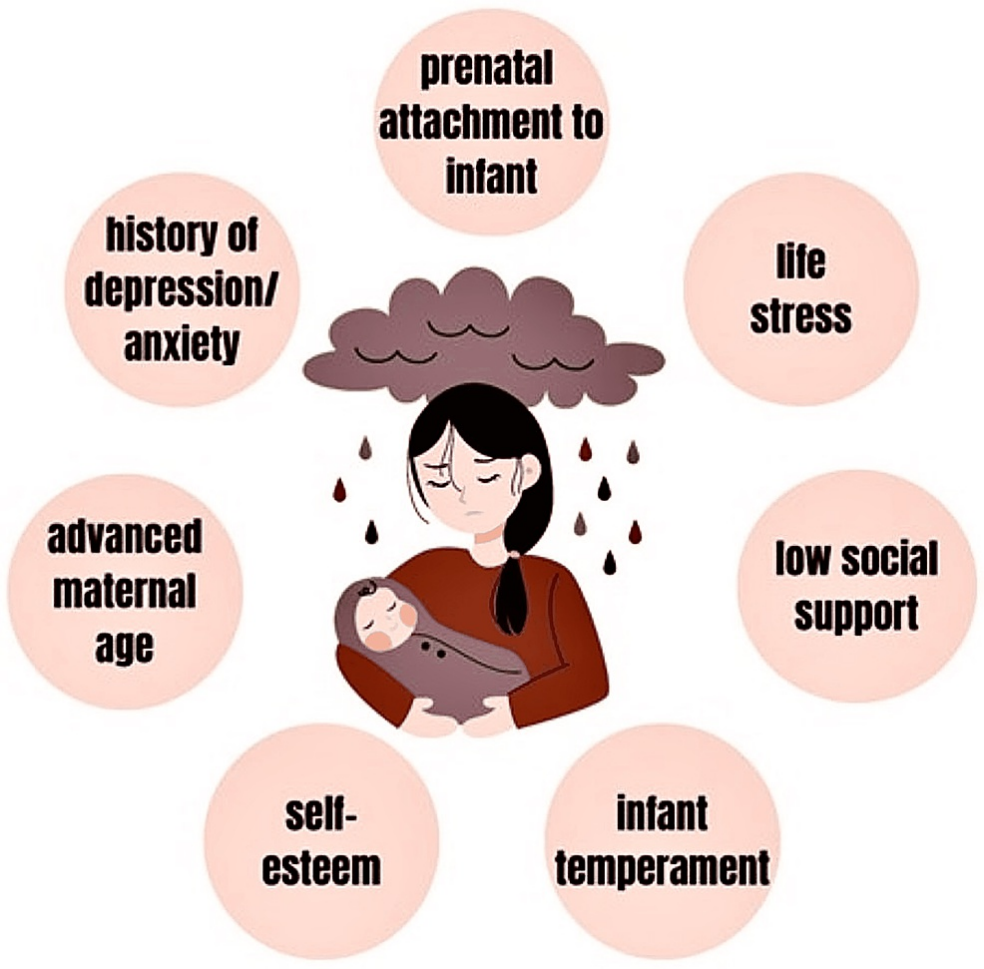
Risk factors for post-partum depression Image Credit: Valerie McAllister (co-author)

Several studies have identified different risk factors for PPD ranging from prior history of depression to low social support. A survey of nulliparous Italian women determined that prenatal attachment to the infant was the strongest predictor of PPD [7]. The other factors contributing to the risk were increased maternal age, quality of the romantic relationship, quality of the mother’s relationship with her parents, and emergency cesarean section. Additionally, prenatal depression or anxiety, self-esteem, childcare stress, life stress, infant temperament, and social support have been identified as significant contributing factors (Figure 1). Socioeconomic status, marital status, and whether the pregnancy was planned or unplanned are additional predictors of postpartum depression [4,6].

PPD creates personal suffering and diminishes a woman's ability to function effectively in many spheres of her life. The added factor of a postpartum woman having significant responsibility for the care of a young infant results in interference with parenting and is associated with a variety of adverse child outcomes in the near and long term [13]. Women with depressive symptoms are less likely to attend well-child visits, complete immunizations, use home safety devices, or place infants in the recommended sleeping position [14].

While a wide range of antidepressants has been evaluated in open and controlled trials in the treatment of PPD, including sertraline, paroxetine, venlafaxine, fluoxetine, nefazodone, and nortriptyline, and several open trials have found that a variety of antidepressants are associated with symptomatic improvement over eight to 12 weeks of treatment, the controlled trials have yielded more equivocal results [13,15]. This has led researchers to look beyond the traditional list of antidepressants. 

One such unconventional candidate is ketamine. First synthesized in 1962 and approved by the FDA as an anesthetic in 1970, ketamine, an N-methyl-D-aspartate (NMDA) receptor antagonist, has established its place in both human and veterinary medicine [16,17]. This versatile drug's complexity lies in its dual capacity as both an anesthetic and analgesic. It is recognized for inducing a state of "dissociative anesthesia", where profound analgesia, amnesia, and preserved respiratory and cardiovascular function create a different experience from traditional sedatives and anesthetics [18]. Despite fluctuations in usage due to concerns about psychomimetic effects, ketamine remains a cornerstone in modern anesthetic regimes [19].

Moving beyond the realms of anesthesia, ketamine has demonstrated remarkable analgesic properties even at sub-anesthetic concentrations [20]. Whether administered intramuscularly at less than 2 mg/kg as a bolus dose, intravenously or epidurally at a dosage under 1 mg/kg or as a continuous intravenous infusion at a rate of 20 μg/kg/min or lower, it effectively mitigates pain [6]. This versatility in dosage underpins its potential efficacy in pain management.

In addition to its well-known role as an anesthetic and analgesic, ketamine's potential as an antidepressant has come to light. Sub-anesthetic doses have been observed to induce feelings of euphoria and show promising anxiolytic effects at smaller doses [21]. Studies have demonstrated that a 40-minute infusion of 0.5 mg/kg ketamine can significantly reduce depressive symptoms within 72 hours, broadening the scope of ketamine's therapeutic applications [22].

However, one particular area that seems to be in the shadows is ketamine's use as an anesthetic in obstetric care with potential benefits for PPD. PPD, a predominant mood disorder affecting new mothers, significantly affects mother-infant bonding and the infant's well-being and development. It is reported that about 25% of women experience PPD in the first three months following delivery [22]. Given the potential of effective labor pain management in reducing the incidence of PPD, the dual analgesic and antidepressant properties of ketamine may enhance labor analgesia regimens [23,24]. Despite these promising indications, the research into this multifaceted application of ketamine is strikingly insufficient, indicating the need for more focused studies in this field.

Several studies have shown that ketamine has benefits in improving PPD symptoms. As an intraoperative agent administered within five minutes of cord clamping, it resulted in mothers with EPDS scores greater than 9 one week postpartum [25]. Ketamine administration is also shown to have some protective factors. When administered 10 minutes after a C-section, it was protective against PPD and its associated risk factors, such as suicidal ideations, antenatal depression symptoms, and stress [26].

However, the use of ketamine must be further explored in the use of obstetric anesthesia. As a lipophilic drug, it can cross the placenta; thus, optimal dosing must be determined to ensure the safety of the fetus. In most research, dosing has been maintained at less than 1.5 mg/kg to ensure the safety of the mother and fetus. In animal studies, ketamine has neuroprotective effects such as anti-inflammatory properties and reduction in neuronal loss [27].

Although there are many benefits to the use of the drug, its risks, and other applications need to be further explored. For example, it cannot be used in the setting of pre-eclampsia due to its preservation of systemic vascular resistance and its ability to increase catecholamine circulation, so its use in high-risk patients may be limited [28]. Also, there is not enough information about ketamine and breastfeeding; thus, its use as a long-term agent may not be possible until more research has been done. Also, many of the leading informational studies have primarily been performed in China. Therefore, our research aims to show its application in the United States medical setting.

## Conclusions

Our study presents a noteworthy observation on the potential use of ketamine as an anesthetic during labor and its potential benefits in mitigating PPD. While this case provides a preliminary and individual-specific insight into the potential benefits of ketamine, it is necessary to underscore the need for randomized controlled trials to confirm these observations. As our understanding of PPD broadens, the inclusion of novel therapeutic agents like ketamine may shift the paradigms of both obstetric anesthetic care and postpartum mental health management. Finally, our study prompts healthcare providers to recognize the profound influence of childbirth pain management on postpartum mood disorders, urging a comprehensive and multi-faceted approach to the overall well-being of both mother and child.
